# Diversity among Clients of Female Sex Workers in India: Comparing Risk Profiles and Intervention Impact by Site of Solicitation. Implications for the Vulnerability of Less Visible Female Sex Workers

**DOI:** 10.1371/journal.pone.0073470

**Published:** 2013-09-02

**Authors:** Dipak Suryawanshi, Tarun Bhatnagar, Sucheta Deshpande, Weiwei Zhou, Pankaj Singh, Martine Collumbien

**Affiliations:** 1 Tata Institute of Social Sciences, Mumbai, India; 2 National Institute of Epidemiology (ICMR), Chennai, India; 3 National AIDS Research Institute, (ICMR), Pune, India; 4 London School of Hygiene and Tropical Medicine, London, United Kingdom; UCL Institute of Child Health, University College London, United Kingdom

## Abstract

**Background:**

It seems generally accepted that targeted interventions in India have been successful in raising condom use between female sex workers (FSWs) and their clients. Data from clients of FSWs have been under-utilised to analyse the risk environments and vulnerability of both partners.

**Methods:**

The 2009 Integrated Biological and Behavioural Assessment survey sampled clients of FSWs at hotspots in Andhra Pradesh, Maharashtra and Tamil Nadu (n=5040). The risk profile of clients in terms of sexual networking and condom use are compared across usual pick-up place. We used propensity score matching (PSM) to estimate the average treatment effect on treated (ATT) of intervention messages on clients’ consistent condom use with FSW.

**Results:**

Clients of the more hidden sex workers who solicit from home or via phone or agents had more extensive sexual networks, reporting casual female partners as well as anal intercourse with male partners and FSW. Clients of brothel-based sex workers, who were the least educated, reported the fewest number/categories of partners, least anal sex, and lowest condom use (41%). Consistent condom use varied widely by state: 65% in Andhra Pradesh, 36% in Maharashtra and 29% in Tamil Nadu. Exposure to intervention messages on sexually transmitted infections was lowest among men frequenting brothels (58%), and highest among men soliciting less visible sex workers (70%). Exposure had significant impact on consistent condom use, including among clients of home-based sex workers (ATT 21%; p=0.001) and among men soliciting other more hidden FSW (ATT 17%; p=0.001). In Tamil Nadu no impact could be demonstrated.

**Conclusion:**

Commercial sex happens between two partners and both need to be, and can be, reached by intervention messages. Commercial sex is still largely unprotected and as the sex industry gets more diffuse a greater focus on reaching clients of sex workers seems important given their extensive sexual networks.

## Introduction

Female sex workers (FSWs) are considered a “core” group for HIV transmission because of high infection rates and large numbers of sexual partners [1]. Their clients then act as a “bridge” population, linked into a larger sexual network with spouses, girlfriends and casual partners who are generally considered at low risk of infection [2–8]. In India, adult HIV prevalence was 0.31% in 2009, and most HIV transmission is through heterosexual networks accounting for 88% of the total reported HIV cases [9] Nearly two thirds (57%) of HIV infections occur in four contiguous states in the South (Andhra Pradesh, Karnataka, and Tamil Nadu) and West (Maharashtra) [10]

Both interventions and research is more extensive among FSWs because they are easier to reach, even if the context is complex. There is great diversity in typology of FSWs at national and state level in India [11]. Studies use various criteria to define type of female sex work based on ‘place of sex’ [12,13] and ‘primary place of solicitation’ [14,15]. In a review, the place of solicitation of sex workers seemed the most suitable criteria to classify FSWs for programming purposes, but the authors acknowledge that it is still largely unknown how this captures the variability in risk and vulnerability of sex workers [11]. Since the scale-up of targeted intervention, several studies have documented a shift towards a more diffuse form of sex trade, with women selling sex from home and soliciting sex via mobile phones, with or without the help of agents who may also make sex workers available at hotel and lodges [11,16–18]. There has been a decline in the more visible forms of sex work solicitation on the streets and at brothels and little is known about how this influences risk reduction in commercial sex.

The size of both the FSW population and their client group, and the rate of unprotected sexual contacts between them are important determinants of the rate of HIV transmission [19]. High levels of sexual risk behaviours have been reported among male sub-populations like attendees of sexually transmitted disease (STD) clinics [20], homeless men [21], contract migrant workers [22] and truckers [23]. Using nationally representative behavioural surveillance data from 2006, Gaffey et al [24] estimate that 8.5 million Indian men had sex with a female sex worker in the last year. In the high HIV prevalence states, 8% of sexually active men reported commercial sex in the last year, compared to only 3% in the low prevalence states.

The first Integrated Bio-Behavioural Assessment (IBBA) survey in Andhra Pradesh, Maharashtra and Tamil Nadu, showed that 28% of clients reported ‘every time’ condom use with sex workers [7]. A quarter of the clients (27%) reported commercial partners only, 47% reported also having a main sexual partner and 27% reported casual and main female partners [7].

Early HIV prevention efforts in India have targeted clients of FSWs through HIV/AIDS awareness campaigns for general population and for truckers through the ‘Healthy Highways’ program [25]. The coverage of bridging populations especially truckers and migrants was expanded under phase III (2006-2011) of India’s National AIDS Control Program (NACP-III) with a strong focus on condom social marketing, STI and HIV testing, treatment and care services [26]. Avahan, the India AIDS Initiative of the Bill and Melinda Gates Foundation, reached out to men visiting FSWs in two main ways. Men were targeted at ‘hotspots’ where clients were most likely to look for sex. The aim was to improve consistent condom use and to create demand for, and access to, STI services [27]. From November 2004 information on HIV, STI treatment, safe sex and condoms was disseminated via a variety of mid-media activities including drama, street theatre, and small group discussions in 100 priority towns in the four high prevalence states [27,28]. Access to condoms was increased by using non-traditional out-lets frequented by men including betel leaf and tobacco shops. During 2007 and 2008 men in the general population were also targeted by a mass-media campaign via television, radio and online public service advertisements designed to normalise condom use as smart and responsible [29].

In this paper we document the profile of clients, during the second round of IBBA surveys in 2009, in terms of demographics, sexual networking and condom use across different solicitation points: from the more visible (public place/brothel) to the less visible (home -based or via phone/agents). We thus indirectly assess sex workers’ vulnerability by typology. We use propensity score matching (PSM) to estimate the impact of intervention messages on clients’ consistent condom use with FSW.

## Methods

### Ethics statement

The Integrated Bio-Behavioural Assessment was approved by the ethical review boards of all participating institutes viz. Indian Council for Medical Research (ICMR), Family Health International (FHI) and the screening committee, Health Ministry, Government of India. A written informed consent was obtained from all the participants at study entry. All consented individuals were given a unique identification number. Behavioural and biological information was linked anonymously to safe guard the participant’s right. Consented participants were given monetary compensation for their efforts and time. This in-depth secondary analysis was approved by the ethics review board of the London School of Hygiene and Tropical Medicine.

### Study settings, sampling design and participant recruitment

The data used in this study is derived from the conducted in India’s high HIV burden states among male clients of FSWs (“clients”) during the year 2009. The participants were recruited from 5 districts in Andhra Pradesh (East Godavari, Guntur, Hyderabad, Visakhapatnam and Warangal), 4 districts in Maharashtra (Mumbai, Parbhani, Pune and Yevatmal) and 3 districts in Tamil Nadu (Chennai, Madurai and Salem). The survey was not done among clients in Karnataka in the same year and the analysis is thus restricted to three states.

An18 to 60 years old man who had paid a woman for sex in the previous month was considered a “client”. Time location cluster (TLC) sampling approach was used to capture different types of clients at selected primary sampling units (PSUs) where FSWs were sampled [30]. The PSUs included solicitation sites for FSWs such as streets, homes, brothels/brothel areas and lodges. Nearly 400 (371–408) clients were sampled from each selected district. A total of 9,808 were approached for participation in the survey, 5,045 completed the interview (response rate of 51%) while 4803 also provided biological samples (response rate 49%). A total of 5040 eligible clients were included in this analysis, after dropping five who did not fulfil the selection criteria.

The clients were interviewed face-to-face in local language by trained investigators in a private setting away from the solicitation site followed by blood draw for HIV testing. For further details of the IBBA methodology see [7,30,31].

### Variables

We could not use biological outcomes in this analysis: it is conceptually incorrect to link a life-time infection like HIV to intervention exposure in the last six months; and the prevalence of acute STIs including *Neisseria gonorrhoeae*, *Chlamydia trachomatis* and acute syphilis was too low (5.1%) to be used as an outcome. Hence, we considered individual protection against HIV/STIs measured by “consistent condom use with *all* sex workers in the last six months” as the outcome variable. The survey instrument distinguishes occasional sex workers (those whom they visited only once or twice but they would not recognise them) from regular ones (those they visited repeatedly and are known to them) and condom use was reported separately for both types of sex workers. A client was considered to consistently use condoms when they reported no unprotected sex acts based on responses to six different questions: a) ‘every time’ use with regular FSW AND b) ‘every time’ use with occasional FSW, AND c) used condom at last sexual intercourse with regular FSW AND d) used condom at last sexual intercourse with occasional FSW, AND e) ‘every time’ use during anal sex with FSWs, AND f) confirming there has not been even one occasion when he did not use a condom during anal or vaginal sex with a regular or occasional FSW.

We considered two measures of exposure: a) Heard or seen messages/advertisements on ‘Condoms’ in the past six months; and b) heard or seen messages/advertisements on ‘STI’ in the past six months. The very high exposure to condom messages however precluded the analysis of its impact.

We included background characteristics that could influence both the exposure and outcome, such as age(/age groups), age at first sex, age at first paid sex, cohabitation, education status (illiterate, primary, secondary+), frequency of travelling outside the current place of residence (not at all, once/twice in a year, once every four to six months, once every two to three months, more than four times in a month), bought sex while travelling, number of different FSW partners in the past six months, anal sex with FSW in the past six months, sex with non-paid casual female partner in the past one year, and alcohol consumption.

### Statistical analysis

All analyses were stratified by ‘usual pickup place of FSW’ (public place/brothel/home/other). The other category includes telephone (60%), hotel/lodge (20%), agent (13%) and bars/night clubs. We described the characteristics of the clients and compared the proportions using chi-square test and medians using k-sample median test. Bivariate analyses show the association of the exposure with consistent condom use (using chi-square test) by solicitation site. We applied the propensity score matching (PSM) technique [32–34] to control for the measured background characteristics and calculate the propensity score for each individual. Individuals with similar propensity score were matched using the radius matching method [35]. The PSM models were built separately by the usual pickup place of FSWs with ‘state’ included as a covariate. However, state-specific models were built, separately, for the clients soliciting at public places and homes as the overall model did not achieve balance for the covariates available in the data. We estimated the Average Treatment effect on those Treated (ATT) as the difference in consistent condom use amongst the exposed (seen or heard messages/advertisements) and the matched unexposed [32]. We calculated bootstrapped standard errors around ATT to calculate the p-value and 95% confidence interval [34]. We checked the assumptions underlying the PSM procedure [34] using the <pscore> and <pstest> command in STATA. We used STATA version 11.0 for all analyses.

## Results

### Socio-demographic characteristics of clients

Of the 5040 male clients included in our sample, 50% mentioned public places as usual pick-up place, 34% brothels, 8% homes and 9% usually solicited using other channels. As shown in Table 1, the median age of the clients was 29 years (IQR 11 years). Overall 72% clients were educated up to secondary level or above and nearly half of all the clients (47.5%) were labourers. Age, education and occupation were highly associated with usual pick-up place (p<0.001). Clients usually picking up FSWs from public places were older than others. More clients soliciting brothel-based FSWs were illiterate or educated up to primary level (40%), compared to those soliciting at all other places (< 25%). The highest proportion of labourers was found among clients soliciting at public places (55.4%) while among clients soliciting home-based FSWs, 46% were either self-employed or salaried.

**Table 1:** Socio-demographic characteristics of male clients of FSWs by usual pickup place, IBBA-2, 2009.

Demographic Characteristics		Usual pickup place of female sex worker				
	Total	Public	Brothel	Home	Other	p value
		Places				(Wald-chi
	[N=5040]	[n=2517,50%]	[n=1703, 34%]	[n=390,8%]	[n=430,9%]	square
	%	%	%	%	%	test)
*Current age [years]*						
Median Age [IQR]	29 [11]	30 [11]	28 [11]	29 [11]	27 [9]	< 0.001*
*Educational attainment*						< 0.001
Illiterate	21.9	19.4	27.4	17.3	15.9	
Primary school (1^st^-4^th^ std.)	6.5	3.2	12.2	2.3	2.2	
Secondary School (5^th^-10^th^ std.)	51.4	57.2	44.9	50.6	52.0	
Secondary+ (above 10^th^ std.)	20.2	20.2	15.5	29.8	30.9	
*Occupation*						< 0.001
Labour	47.5	55.4	43.7	33.0	38.6	
Self-employed/Large businessmen	17.1	10.3	24.7	22.6	13.6	
Salaried	14.1	11.2	15.3	23.0	15.5	
Transport/ Bus workers	13.8	16.6	11.5	9.0	12.3	
Not earning	5.6	4.6	3.9	6.6	16.8	
Others	1.9	1.9	0.9	5.8	3.2	
*Current Marital Status*						0.0047
Never married	34.9	30.5	39.6	27.1	43.9	
Currently married	60.5	65.1	57.2	64.1	48.1	
Separated / Divorced / Widowed	4.6	4.4	3.2	8.7	8.0	
*Having main/steady partner*	67.1	70.1	65.3	73.1	54.4	0.0129
*Currently cohabiting*	59.1	66.5	50.0	70.0	51.9	< 0.001
*State*						< 0.001
Maharashtra	42.8	11.2	92.3	2.1	21.6	
Andhra Pradesh	36.6	46.6	7.2	92.0	64.4	
Tamil Nadu	20.6	42.3	0.6	6.0	14.1	

* k-sample median test; IQR: interquartile range

Overall 61% of clients were currently married, 67% reported a steady sexual partner and 59% were cohabiting with a sexual partner. Cohabitation status was more strongly correlated with usual pick-up place (p<0.001) than marital status and reporting a steady partner (p< 0.05). The highest proportion (44%) of “never married” clients was amongst those soliciting via phone or other venues. Compared to other groups, fewer clients soliciting brothel-based FSWs were cohabiting (50%): among those married 16% reported living away from their spouse, compared to 4% among married clients soliciting elsewhere (not shown).

Solicitation sites vary significantly by state (p<0.001). Ninety two percent of clients soliciting at brothels were from Maharashtra. Among the clients picking up home-based sex workers 92% were from Andhra Pradesh. Of the clients soliciting at public places 42% were from Tamil Nadu. The six brothel-based clients from Tamil Nadu were dropped from subsequent analyses.

### Sexual partners, sexual practices and condom use

Table 2 presents data on the prevalence of different sexual partners, condom use and exposure to prevention messages. Median age at first sexual intercourse was 19 years [IQR 3 years] and median duration of soliciting sex work was 7 years [IQR 3]. In all, 66% of men reported frequenting ‘regular’ FSWs (ranging from 52% among those soliciting at brothels to 77% among those at public places; p<0.0001), while 91% reported sex with ‘occasional’ FSWs in the past six months with no significant variation across solicitation sites. Fifteen% of men reported anal sex with male or hijra (transwoman) partner in the last six months (from 8% among those picking-up at brothels to 21% among home-based and others; p<0.0001) and 14% reported anal sex with FSWs (from 5% among brothel-based to 20% at public places; p<0.0001).

**Table 2:** Sexual practices and partners, consistent condom use and exposure to interventions among male clients of FSWs by usual pickup place, IBBA-2, 2009.

		Usual pickup place of female sex worker				
	Total	Public	Brothel	Home	Other	p value
		Places				
	[N=5034]	[n=2517]	[n=1697]	[n=390]	[n=430]	(Wald-chi
	%	%	%	%	%	square test)
**Sexual practices and partners**						
Median age at first sexual intercourse (years) [IQR]	19 [3]	19 [3]	19 [3]	18 [3]	19 [3]	0.007*
Median duration of paid sex (years) [IQR]	7 [9]	7 [3]	7 [9]	7 [9]	5 [8]	0.001*
Had sex with occasional FSW in past 6 months	91.0	92.1	88.2	95.1	92.5	0.132
Had sex with regular FSW in past 6 months	66.1	76.8	51.5	76.5	66.7	< 0.0001
Number of FSWs in past 6 months [IQR]	6 [6]	7 [7]	4 [5]	6 [6]	7 [6]	< 0.0001*
Had sex with non-paid casual female partner in past one year	16.1	16.0	11.6	31.9	21.1	< 0.0001
Had anal sex with FSW in past 6 months	13.9	20.3	5.4	16.9	16.0	< 0.0001
Had anal sex with male or hijra partner in past 6 months	15.2	18.9	8.5	20.7	21.0	< 0.0001
**Condom use**						
Consistent condom use with occasional sex	53.8	51.9	46.9	81.8	65.3	< 0.0001
workers	[n=4655]	[n=2326]	[n=1548]	[n=369]	[n=412]	
Consistent condom use with regular sex	44.9	44.1	35.7	68.6	55.6	0.0001
workers	[n=3428]	[n=2027]	[n=810]	[n=284]	[n=307]	
Consistent condom use with all sex workers	45.2	41.7	41.4	67.0	59.0	< 0.0001
Consistent condom use with casual female	27.1	23.1	56.3	25.7	30.6	0.0006
partners	[n=900]	[n=439]	[n=246]	[n=101]	[n=114]	
Consistent condom use with main female	2.1	0.7	2.1	8.3	3.9	0.0005
sexual partner	[n=3314]	[n=1696]	[n=1103]	[n=256]	[n=259]	
Consistent condom use with male/hijra partner	37.0 [n=752]	36.5 [n=448]	19.2 [n=184]	66.6 [n=43]	50.5 [n=77]	0.0072
**HIV prevalence**	6.5 [n=4789]	6.9 [n=2398]	6.3 [n=1607]	9.6 [n=377]	3.0 [n=407]	0.396
**Exposure to interventions**						
Heard or seen advertisement/messages on 'Condom' in past 6 months	94.4	92.7	97.7	87.7	94.8	0.0007
Heard or seen advertisement/messages on 'STI' in past 6 months	68.8	77.6	58.2	70.0	70.0	< 0.0001

* k-sample median test; IQR: interquartile range

Of the 390 clients who solicited home-based FSWs, 77% and 95% reported regular and occasional sex workers, respectively in the last six months, 32% reported non-commercial casual partners in the last year and 21% reported anal sex with men or hijras in the last 6 months. In contrast, clients soliciting brothel-based FSWs showed least sexual mixing: the lowest proportion of reporting sex with regular (52%) and occasional (88%) sex workers, casual female partners (12%), male/hijra partners (9%), and anal sex with FSWs (5%).

Overall, 45% clients reported consistent condom use with all FSWs, ranging from 41% for clients soliciting at brothels to 67% for clients of home-based FSWs (p<0.0001). Among the clients soliciting at brothels, 36% reported consistent condom use with regular sex workers (n=810) which was lower than that reported by home-based soliciting clients (69%, n=284) and other FSWs (p<0.0001). Consistent condom use with occasional FSWs was highest among clients soliciting home-based FSWs (82% compared to an average of 54%; p=0.001). In all, 37% of 752 clients reported consistent condom use with male/hijra partners, ranging from 19% among those soliciting FSWs at brothels to 67% soliciting home-based FSWs (p=0.0072). Consistent condom use with casual female partner reported by 27% of 900 clients ranged from 23% among the clients soliciting FSWs at public places to 56% among those soliciting at brothels (p=0.0006) (Table 2).

Conversely, clients who frequented home-based sex workers did report the highest levels of condom use with all categories of partners except for casual female partners. They were more likely to report a main sexual partner (Table 1) and 8% reported consistent condom use with them (compared to 2% for all clients). Overall 6.5% of 4795 clients tested were HIV positive, ranging from 3% (others)-10% (home-based) (p=0.396). HIV status did not vary significantly by state, nor was it statistically associated with any of the consistent condom use measures, extent of sexual networks, practice of anal sex or exposure to interventions (not shown).

Overall, 94% of all clients reported having heard or seen advertisements/messages about condoms, with the highest among those soliciting at brothels (98%). In all, 69% of clients reported being exposed to messages on STIs, with the lowest among those soliciting at brothels (58%).

### Consistent condom use with female sex workers by exposure to STI messages in different states

Overall, exposure to STI messages was reported by 62% clients in Maharashtra (MH), 65% in Andhra Pradesh (AP) and 90% in Tamil Nadu (TN). Consistent condom use with FSWs was reported by 29% in Tamil Nadu, 36% in Maharashtra and 65% clients in Andhra Pradesh (Table 3).

**Table 3:** State-wise exposure to STI messages and consistent condom use with sex workers among the clients by usual pickup place, IBBA, 2009.

State		Usual pickup place of female sex worker			
	Total	Public	Brothel	Home	Other
		Places			
	%	%	%	%	%
**Andhra Pradesh**	[n=2080]	[n=1270]	[n=182]	[n=358]	[n=270]
*Heard/seen advertisement/messages on 'STI' in past 6 months*	64.6	63.2	57.2	67.9	69.0
*Consistent condom use*					
among all	64.7	61.6	60.7	70.0	71.2
among unexposed	54.9 [n=696]	54.5 [n=422]	44.7 [n=75]	61.2 [n=118]	55.2 [n=81]
among exposed	70.1 [n=1384]	65.7 [n=848]	72.7 [n=107]	74.1 [n=240]	78.4 [n=189]
p-value (Wald-chi square test)	0.0002	0.0264	0.0178	0.1901	0.0253
**Maharashtra**	[n=1674]	[n=94]	[n = 1515]	[n=6]*	[n=59]
*Heard/seen advertisement/messages on 'STI' in past 6 months*	62.4	86.5	58.3		75.4
*Consistent condom use*					
among all	36.4	17.7	39.6		27.6
among unexposed	33.9 [n=800]	14.9 [n=32]	35.3 [n=742]		19.5 [n=23]
among exposed	37.8 [n=874]	18.2 [n=62]	42.7 [n=773]		30.3 [n=36]
p-value (Wald-chi square test)	0.50	0.786	0.265		0.608
**Tamil Nadu**	[n=1280]	[n=1153]		[n=26]*	[n=101]
*Heard/seen advertisement/messages on 'STI' in past 6 months*	89.8	91.1			66.1
*Consistent condom use*					
among all	28.7	26.0			51.0
among unexposed	48.7 [n=174]	43.0 [n=131]			67.0 [n=41]
among exposed	26.4 [n=1106]	24.4 [n=1022]			42.8 [n=60]
p-value (Wald-chi square test)	0.0005	0.007			0.137

* *sub-sample too small for meaningful comparison*

On bivariate analysis, 70% of exposed clients reported consistent condom use with FSWs compared to 55% among unexposed (p = 0.0002) in Andhra Pradesh. The association was statistically significant among all groups except for clients of home-based sex workers. Overall, there was no significant association between consistent condom use with FSWs and exposure to STI messages in Maharashtra, where 90% of 1674 clients solicited brothel-based FSWs. In Tamil Nadu, where 90% of 1280 clients solicited sex workers at public places, consistent condom use with FSWs was reversely associated with exposure (p = 0.0005). Among the clients soliciting at public places, who had heard/seen STI messages in the last 6 months, condom use was 24% compared to 43% among the 9% of unexposed clients (p=0.0007).

Table 4 shows the impact of exposure to ‘STI advertisements/messages” on the clients’ consistent condom use with FSWs adjusted for background characteristics using propensity score matching. Among the clients usually picking up sex workers at public places in Andhra Pradesh, consistent condom use with FSWs was 17% higher among those exposed to STI messages compared to the matched controls (p<0.001). In Tamil Nadu, the negative association between exposure and condom use could not be confirmed by propensity score matching as we failed to get a balanced model. This means that unknown confounding factors beyond the covariates measured in the data affect the difference in condom use between the exposed and unexposed men (and hence matching was inadequate). Among the clients soliciting at brothels in Maharashtra, consistent condom use with FSWs was 39% for the clients unexposed to STI messages and 49% for the exposed (ATT=10%, p<0.001). When the sample is limited to the 328 clients sampled near Mumbai brothels the ATT was as high as 28% (p<0.001). No significant effect could be shown among clients frequenting brothels in Andhra Pradesh, but for clients of home-based sex workers there was a 21% difference in condom use between those exposed to STI messages compared to their matched controls (p=0.001). For the combined sample of clients soliciting at other places or by phone, the effect of exposure was 17% (p=0.001).

**Table 4:** Average Treatment Effect on Treated (ATT) for consistent condom use with all sex workers among male clients of female sex workers by usual pickup place of FSW, IBBA, 2009.

Usual pick-up place of FSWs (State)		Consistent condom use (%) among				
	N	Treated (heard/ seen STI messages)	Matched controls (not heard/ seen STI messages)	ATT (%)	Bootstrap S.E.	p-value
Public Place (AP)	1270	71.7	55.2	16.5	0.03	< 0.001
Brothel (AP)	182	68.7	64.1	4.6	0.08	0.567
Brothel (MH)	1515	48.5	38.9	9.6	0.03	<0.001
Home-based (AP)	358	78.6	57.3	21.3	0.07	<0.001
Other places (AP/MH/TN)	430	63.1	46.0	17.1	0.05	<0.001

AP: Andhra Pradesh; MH: Maharashtra; TN: Tamil Nadu

## Discussion

While targeted interventions among FSWs in India have been widely studied, our analyses focuses on the under-researched group of the clients of these sex workers and how their risk profile differs according to the place of solicitation. Heterogeneity is evident in their socio-demographic profile, the extent of their sexual networks, consistency of condom use, exposure to intervention messages and impact on levels of protection. Those usually accessing sex workers from public places and from brothels were contrasted to those using sex services from less visible home-based sex workers or in the last category women contacted via the phone or operating through agents, at hotels/lodges, bars and night clubs. Clients in this last category tended to be younger, more likely to be single, and started buying sex on average only 5 years ago compared to 7 years among all clients. This group and men soliciting home-based sex workers were significantly better educated, especially when compared to clients of brothel based sex workers. Men visiting brothels were least likely to cohabit with a sexual partner, and more likely to live away from their spouse.

The extent of the clients’ sexual networks, the riskiness of sexual practice they engage in and the risk reduction behaviour they adopt combine to affect their own risk and that of their partners: commercial and non-commercial, male and female. Overall, men reported nearly as many male or hijra partners in the last 6 months (15%) as casual female partners in the last year (16%). Clients of FSWs thus form an important bridge between several groups at higher risk (FSW, MSM and hijras) and to casual and marital partners.

Men visiting brothels stand out as reporting fewer casual and commercial female partners and less anal intercourse with men/hijras and FSWs compared to other clients groups. However, they did report the lowest levels of consistent condom use with sex worker (occasional and regular) and with male and hijra partners. Only with casual female partners was condom use higher than other client groups. Men soliciting sex-workers at public places had more extensive sexual networks than brothel-based sex workers and condom use levels were only marginally higher.

Consistent with their higher educational status, men soliciting the more hidden sex workers did report higher levels of condom use, not only with sex workers but also with male and hijra partner. However, a larger proportion engaged in anal sex either with FSW (16-17%) or with male/hijra partners (21%). Sexual networking was most extensive among men who solicited home-based sex workers, with nearly a third reporting casual female partners. While the different components contributing to the risk of HIV transmission varied significantly across these four diverse groups of clients, they combined into HIV prevalence rates that were not significantly different.

The low levels of protection in commercial sex deserve further discussion. The overall level of 45% consistent condom use varied more by state of residence than by place of solicitation. Clients in Andhra Pradesh reported consistent condom use at 65%, compared to 36% in Maharasthra and 29% in Tamil Nadu. This is substantially lower than what was measured in November 2008 in the end line survey which evaluated the Avahan-funded component of integrated behaviour change programme targeted at clients implemented by Population Services International (PSI) [28]. Consistent condom use was 76% in Andra Pradesh, 95% in Maharashtra and 79% in Tamil Nadu. Factors to explain such divergent levels include 1) eligibility criteria (men reporting commercial sex in the last 12 months (PSI) versus in the last month (IBBA), 2) recruitment (data collection during daylight (PSI) while IBBA carried on at night-time) and 3) most importantly the sampling strategy. The PSI survey [28] aimed for a representative sample of men who buy sex at the same hot spots where PSI implemented a mix of interpersonal communication and mid-media activities supported by static outdoor promotional materials. Sampled at the end of an intense campaign of nearly 4 years, it is inherently biased towards men with highest intervention exposure. Clients in the 2009 IBBA are more representative of all men who buy sex at randomly selected hotspots, with less intense intervention exposure on average, and consequently less affected by social desirability bias. Hence there seems no reason to doubt the validity of the clients’ condom use levels reported in this study.

In the 2009 IBBA, 85% of the sex workers reported consistent condom use with occasional and regular clients: 82% in Andhra Pradesh, 95% in Maharashtra and 87% in Tamil Nadu [36]. These levels are not borne out by what clients reported. While clients were sampled in fewer districts than the FSWs one would expect more agreement between the estimates. Social desirability bias is likely to have inflated reports by FSWs since they have been so intensely targeted by interventions on an almost daily basis. On the contrary, exposure to intervention for clients was far less intense, less frequent and less based on interpersonal communication making their responses probably nearer to the truth. Trends in HIV prevalence in Tamil Nadu indeed lend more credibility to reports from men as prevalence among clients was higher in the second round (0.7%-10.2% in 2009 compared to 2%-4.2% in 2006 vis-a-vis that among sex workers that did not decline but remained stable at 6.1% between 2006 and 2009 [36–38]. Furthermore, HIV prevalence in Maharashtra increased among FSWs from 26% to 27.5% which was statistically significant among street-based FSWs in Mumbai and brothel-based FSWs in Thane districts [36,39].

The most visible sex workers (brothel and street-based) are the most intervened, and the most monitored and surveyed group. They have been the focus of most published papers discussing targeted interventions in India which have repeatedly pointed to the gains in protecting commercial encounters [40–43], while all along acknowledging the potential for desirability bias [36,42–45]. These levels are quite contrary to those reported with other partners [36,44,46,47]. . This has lead to an implicit assumption that the main problem now lies in unprotected sex with intimate and regular partners [48]. The second emerging concern is that the sex trade is becoming increasingly hidden, with larger proportions of sex workers operating in less visible ways including via mobile phones [11,17,18]. These sex workers are less easy to reach with interventions as well as research. By definition clients of sex workers are difficult to identify and thus difficult to reach with interventions and surveys. Thus an encouraging finding is that 70% of men soliciting these less visible sex workers (home-based and the remaining ‘other’ category) had been reached with messages on STIs. Presumably this exposure was mainly through mass-media. At brothels, where the potential for more interactive communication channels is highest, exposure to STI messages was lowest (58%; even though 98% had received messages on condoms). After matching clients on education, occupation and other important covariates, the impact of having received messages on STIs on consistent condom use was lower among clients of brothel-based sex workers than among men frequenting the more hidden home-based sex workers and those soliciting via phone, at lodges or in bars. The impact among clients soliciting at public places in Andhra Pradesh was also high in contrast to Tamil Nadu where campaigns seemed ineffectual. While clients reported highest exposure to messages, those exposed were least likely to use condoms consistently. This is difficult to explain and our PSM analysis confirmed a bias caused by unmeasured factors in Tamil Nadu. These may include more prevention fatigue compared to other states, and a potential adverse effect of propaganda about the free availability of antiretroviral treatment in Tamil Nadu.

Several study limitations need consideration. First, caution is needed in comparing the risk profiles of clients at various solicitation sites, as this may be biased by over and under-sampling of certain groups in particular states. While Tamil Nadu has very little brothel-based sex work [7,17], the vast majority of brothel clients were sampled in Maharashtra where street-based sex workers were under-represented. Most clients of home-based sex workers were in Andhra Pradesh as this was the only state where clients were sampled at solicitation sites for home-based sex work [30]. Indeed, all state-specific samples under-represent clients of hidden sex workers. Secondly, we cannot claim to have a representative sample of clients of the home-based and the ‘other’ sex workers. Men who reported usually soliciting these less visible FSWs were in fact recruited at hotspots or near brothels and may have a different risk profile from those who exclusively solicit hidden sex workers. A third limitation that potentially affects the generalisability of our findings is that nearly half of the men approached and eligible for interview refused to participate. Response rates were higher than in IBBA–I, ranging from 24% to 53% between districts [7] and 37% in the PSI endline survey [28]. While inevitable, and the bias introduced unknown, it seems unlikely that non-responders would have either much lower or much higher risk profiles. Fourthly, the survey has poor measures of exposures with no detail on intensity or communication channel and no ability to attribute exposure to any specific campaign. Finally, social desirability bias in reporting desirable behaviour cannot be ruled out, though as discussed above the extent is probably minor. This is generally a problem in interviewer administered face-to-face surveys related to sensitive issues which can be mitigated using audio computer-assisted self interviews, as successfully applied in Indian settings [49].

Our analysis has important implications for HIV prevention programmes in India. Although it has been strongly argued that it is more effective to focus HIV prevention efforts on sex workers rather than on their commercial partners [24], the balance has probably swung too far. In rapidly changing context where sex workers operate in more covert ways and they themselves become more difficult to reach, recommendations still seem to exclusively focus on targeting FSWs by extending interventions to lodges for example 17. We challenge this neglect of targeting men who buy sex and who have extensive networks with other male and female partners. In this study, men reached with intervention messages are more likely to use condoms (with the important exception in Tamil Nadu) and clients who solicit the more hidden sex workers had been exposed to messages. Considering that clients are such a diffuse group and they do not restrict their partners to sex workers, mass-media campaigns might be the best option to further normalise condom use in all sexual relationships. The high levels of unprotected sex reported by clients show that HIV prevention is still an unfinished business in India and that sex workers too need continued support to increase condom use with all partners, though all responsibility should not rest on them.
